# Time to Refresh: Design and Evaluation of Refresher Training to Sustain Procedural Teaching Skills

**DOI:** 10.5334/pme.2443

**Published:** 2026-05-15

**Authors:** Robert A. Mousset, Wouter H. de Vos tot Nederveen Cappel, Jurjen J. Boonstra, Alexandra M. J. Langers, Paul L. P. Brand

**Affiliations:** 1 Lifelong Learning, Education and Assessment Research Network (LEARN), University Medical Center Groningen, Groningen, The Netherlands; 2 Department of Gastroenterology and Hepatology, Isala Zwolle, Zwolle, The Netherlands; 3 Department of Gastroenterology and Hepatology, Leiden University Medical Center, Leiden, The Netherlands; 4 Department of Medical Education and Faculty Development, Isala Zwolle, Zwolle, The Netherlands

## Abstract

**Background and need for innovation:**

Train-the-trainer courses for procedural teaching, including gastrointestinal endoscopy, have been shown to improve trainer performance and learner outcomes. However, maintaining these gains over time is challenging. Refresher training has been proposed as a strategy to support long-term retention of procedural teaching skills; however, such training is currently lacking and its impact remains unclear.

**Goal of innovation:**

We developed, implemented and evaluated a bespoke Training the Colonoscopy Trainers (TCT) refresher training to support sustained endoscopy teaching skills. In addition, facilitators and barriers influencing long-term retention and transfer of teaching skills were identified.

**Steps taken for development and implementation of innovation:**

Sixteen endoscopy trainers who had previously completed the original TCT course participated in a one-on-one refresher training delivered 6–9 months after the initial training. Effectiveness was evaluated using Kirkpatrick levels 1 (reaction), 3 (behaviour) and 4 (results). Course participant interviews were thematically analysed to identify factors influencing sustained skill retention and transfer to practice.

**Outcomes of innovation:**

Participants highly valued the refresher training. Although improvements in trainer performance were not statistically significant, post-refresher training video-analysis scores exceeded all previous measurements. Residents reported high satisfaction with supervision and perceived improvements in feedback quality and procedural guidance. Institutional support and opportunities to teach were identified as key facilitators, while time constraints were the primary barrier to sustained skill application.

**Critical reflection:**

This study demonstrates the feasibility and added value of refresher training as part of faculty development for procedural teaching. While challenges remain regarding optimal timing and institutional support, this proof of principle highlights the potential of longitudinally embedded refresher training to advance teaching quality across medical education contexts.

## Background and Need for Innovation

There is increasing recognition that healthcare professionals require formal preparation for their educational roles, prompting widespread implementation of train-the-trainer programs [1]. While many programs are generic – focusing on core educational principles such as lesson delivery and providing feedback [1] – the past decade has seen the emergence of programs tailored specifically to procedural teaching. The Training the Colonoscopy Trainers (TCT) course was the first of its kind and successfully improved colonoscopy quality outcomes within the UK’s national colorectal cancer screening program [2]. Building on this success, similar train-the-trainer initiatives targeting different procedures, such as the laparoscopic colorectal (Lapco) TT course, have been developed [3]. Although evaluations of both TCT and Lapco TT have demonstrated significant improvements in trainer performance and resident learning [3,4], sustaining these gains over time remains challenging.

Prior studies on faculty development (FD) initiatives have demonstrated that teaching knowledge and skills may decline considerably over time [5,6]. Factors suggested to influence the sustainability of FD teaching competencies include program design, opportunities for deliberate practice in authentic contexts, and the provision of reinforcement [5,7]. However, it remains unclear to what extent these factors apply within the clinical workplace – a setting more dynamic and unpredictable than the traditional classroom. Refresher training has been proposed as a promising strategy to support the maintenance of procedural teaching [4,8], theoretically informed by Ebbinghaus’ forgetting curve, which suggests that newly acquired skills decline rapidly without timely reinforcement [9]. However, descriptions of such interventions and evidence of their impact are currently lacking. Moreover, program evaluation in FD for clinician-educators remains underdeveloped, with limited use of longitudinal, practice-based measures to assess impact [10,11].

To address these gaps, this paper describes the design, implementation, and evaluation of refresher training for gastrointestinal (GI) endoscopy trainers delivered at two teaching hospitals in the Netherlands. We aim to share practical insights into how refresher training can be embedded in clinical practice and how its impact can be meaningfully evaluated over time.

## Goals of Innovation

The TCT refresher training aimed to enhance the long-term retention of teaching skills among trainers who had previously completed the TCT course [4], thereby promoting consistent, high-quality endoscopy training for residents. In line with previous research [3,4], this study evaluated the effectiveness of the TCT refresher training using Kirkpatrick’s evaluation model [12]. In addition, the study explored key facilitators and barriers influencing the long-term retention and transfer of procedural teaching skills. Although situated in GI endoscopy, this innovation serves as a proof of principle for FD initiatives more broadly, especially those aimed at sustaining procedural teaching skills.

## Steps Taken for Development and Implementation of Innovation

### Course development

The TCT refresher training was developed based on the original TCT course [2] by an expert faculty group from the Leiden University Medical Center, consisting of expert gastroenterologists with many years of experience in TCT course delivery.

A bespoke two-hour, interactive refresher training was designed to reinforce previously acquired endoscopy teaching skills. To address individual learning needs, the refresher training was delivered in a one-on-one format, with a TCT trainer mentoring a single participant. This approach differs from the original TCT course, which is delivered in a group setting [2,4]. The individualized TCT refresher training, referred to from this point as refresher training, comprised four components: (1) a semi-structured interview in which the TCT trainer explored the participant’s endoscopy teaching experiences after the initial TCT course (Appendix 1); (2) a joint review of key theoretical concepts, including the structured training framework, skills deconstruction, and feedback strategies [2]; (3) a hands-on session in which the participant supervised a resident performing a colonoscopy on an actual patient, while being observed by the TCT trainer who was present in the endoscopy room; and (4) a debriefing in which the participant and trainer reflected on the teaching interaction and consolidated key learning points.

This iterative process of experience, reflection, conceptualization, and experimentation aligns with Kolb’s experiential learning cycle [13] and supports both skill refinement and a deeper understanding of effective teaching behaviours. The one-on-one format enabled personalized feedback and goal setting, facilitating the translation of behavioural intentions into sustained teaching behaviour, consistent with the Theory of Planned Behaviour [14].

### Course implementation

Sixteen gastroenterologists from two general teaching hospitals in the northeastern educational region of the Netherlands, all of whom had previously participated in a study evaluating the effectiveness of the original TCT course [4], were invited by email and agreed to participate. Contextual study information is provided in Appendix 2. Between May 2022 and May 2023, four on-site refresher trainings were conducted at the participating hospitals, 6–9 months after the initial TCT courses. All refresher trainings were delivered by AL and JB, who were formally trained to deliver the initial TCT course at St. Mark’s Hospital in the UK and had extensive experience facilitating TCT courses in the Netherlands [4]. Furthermore, AL has extensive experience in developing and delivering a variety of teach-the-teacher courses. As initiators of the program, AL and JB were closely involved in the development of the refresher curriculum. To ensure consistency and high-quality delivery, alignment meetings between AL and JB were held.

### Course evaluation

The interviews at the start of the refresher trainings were video-recorded and transcribed verbatim. To evaluate the effectiveness of the refresher training, Kirkpatrick levels 1 (reaction), 3 (behaviour) and 4 (results) were assessed [12]. In line with previous research [4] and to maintain alignment with the original TCT course format, level 2 (learning) was not evaluated. As an integral part of the refresher training, the hands-on sessions cannot be used as a post-training assessment of learning outcomes. Furthermore, incorporating additional assessment tools would have required extra time, potentially displacing essential course elements and increasing performance pressure during the training, which might negatively affect skill development and participant engagement [4].

- Kirkpatrick Level 1: The initial reaction of participants was evaluated using an online post-training questionnaire (Appendix 3) distributed immediately after the refresher training.
- Kirkpatrick level 3: Changes in trainer behaviour were evaluated through video analysis of endoscopy training sessions in the workplace conducted 1–3 months before (T2) and 1–3 months after the refresher training (T3). Trainer performance was assessed using an adapted version of the validated mini-Structured Training Trainer Assessment Report [15], which has also been used in a previous study [4], and will be referred to as mini-STTAR* (Appendix 4). This itemized scoring system, comprising 31 trainer attributes, evaluates training quality across the Set (preparation phase), Dialogue (training phase), and Closure (wrap-up phase). Each attribute was scored on a 5-point Likert scale (1 – ‘Did not happen but should have been done’; 2 – ‘Happened but not enough’; 3a – ‘Did not happen but did not need to’; 3b – ‘Happened but did not need to’; 4 – ‘Happened perfect amount’).
- Kirkpatrick level 4: Perceived changes in the clinical learning environment were assessed using a post-training resident survey (Appendix 5) distributed six months after refresher training implementation. The 18-item survey – developed by the research team with input from experts in postgraduate medical education – was distributed to all residents in the participating teaching hospitals who performed endoscopies under the supervision of participants. Survey results were compared with data collected from residents in the same hospitals prior to the refresher training [4]. All residents invited to complete the post-refresher training survey had also received the pre-training survey.

### Data analysis

The transcripts of video-recorded semi-structured interviews were analysed qualitatively using an inductive thematic approach [16]. Familiarisation with the data was achieved through repeated and careful viewing of the transcripts. Coding, categorisation, and thematization were performed iteratively by one researcher (RM), with emerging findings discussed within the research team until consensus was reached.

Quantitative descriptive analyses were conducted for Kirkpatrick level 1 and 4 outcomes, with pre- and post-training differences assessed using Pearson’s χ² test. Analyses of video-recorded endoscopy training sessions (Kirkpatrick level 3) followed previously described procedures [4]. Post-refresher training videos (T3) were evaluated in random order together with pre-refresher training videos (T0, T1, and T2), with assessors blinded to the timing of the recordings. As previously reported [4], intra-class correlation analysis demonstrated excellent absolute agreement between raters across the mini-STTAR*, with no evidence of inter- or intra-rater drift. Overall mini-STTAR* scores were computed as the mean of all 31 mini-STTAR* items, and overall Set, Dialogue, and Closure scores as the mean of the items within each respective domain. Pre- and post-refresher training mini-STTAR* scores were compared using non-parametric Wilcoxon signed-rank tests, with effect sizes calculated as \[
r\ =\ \left| {{\textstyle{Z \over {\sqrt N }}}} \right|
\] and interpreted according to Cohen’s guidelines [17]. Statistical analyses were performed using SPSS version 28, with *p*-values < 0.05 considered statistically significant.

### Ethics

The study was approved by the Medical Ethical Committee of Isala Zwolle, the Netherlands (study number: 210603). All participating gastroenterologists and residents provided written informed consent.

## Outcomes of Innovation

### Demographics

The 16 participating gastroenterologists had a mean age of 44.6 ± 6.8 years; five women and eleven men. Their mean experience as an endoscopy trainer was 8.8 years (range 2–19 years).

### Interviews

The mean duration of the interviews conducted at the start of the refresher training was 14 minutes (range 8–22 minutes). Table 1 presents the main themes identified, accompanied with representative participants’ quotes. Participants reflected positively on the initial TCT course and reported applying several elements in their daily teaching practice, particularly the use of a structured training framework, pre-procedural briefings and debriefings, strategies to prevent cognitive overload, and restraint in taking over the endoscope. Becoming a consciously competent endoscopy trainer, as well as formulating clear learning objectives and take-home messages, were most frequently highlighted as areas for improvement. Limited opportunities to train residents in endoscopy and insufficient time for supervision were identified as key barriers to further development as endoscopy trainers.

**Table 1:** Main themes and representative participant quotes from semi-structured interviews at the start of the TCT refresher training.


MAIN THEMES	REPRESENTATIVE PARTICIPANT QUOTES

Use of a structured training framework	“Endoscopy training becomes easier when I follow a structured framework” “It is actually very enjoyable, and I hope that if this approach is widely adopted, residents will consider it a standard part of their endoscopy training”

Pre-procedural briefing and post-procedural debriefing	“We have allocated time before the start of the endoscopy training session to engage with the resident and ask: ‘do you have learning objectives?’ This has now become my standard practice” “I was initially concerned that debriefing would be time-consuming, but it has proven to be manageable. When the learning objective is specific, it can be integrated effectively”

Strategies to prevent cognitive overload	“What I learned from the course is dual-task interference. Recently, during a complex procedure, the nurse and I kept giving instructions to the resident – until I said, ‘Stop, time-out’”

Restraint in taking over the scope	“My own learning objective was: How can I provide the best possible guidance without taking over the scope? […] Since applying the tips from the (initial) TCT course, I have not taken over the scope even once”

Becoming a consciously competent endoscopy trainer	“I still find it difficult at certain moments during a colonoscopy to explain (to the resident) why something is not working. Then I want to take over the scope to understand how I would do it” “I try to be more aware of what I actually do to solve a problem, in order to explain it more effectively to residents”

Specifying learning objectives and take-home messages	“It remains a challenge to determine when a learning objective is sufficiently SMART and not too vague” “I should encourage them (residents) to formulate the take-home message themselves. […] I tend to provide too much input myself”

Limited number of endoscopy training sessions	“Sometimes one or two (endoscopy training sessions per week), other times none for weeks. That makes it quite challenging to fully develop proficiency (in endoscopy teaching)”

Lack of time for endoscopy supervision	“If you have your own endoscopy schedule alongside supervision, it can be very frustrating. […] You tend to solve the problem quickly, which is not beneficial for the resident’s learning”


### Kirkpatrick level 1

The post-training questionnaire was completed by 12 participants (75% response); three women and nine men. Respondents reported that the initial TCT course improved multiple aspects of their endoscopy training practice (Appendix 6). All respondents perceived participation in the refresher training following completion of the initial TCT course as valuable, and 83% believed it should become standard practice. In the free-text responses, 42% noted that the learning objectives achieved during the initial TCT course tend to diminish over time, underscoring the importance of reinforcement, and 25% highlighted the value of the one-on-one training format.

### Kirkpatrick level 3

**Figure 1:**
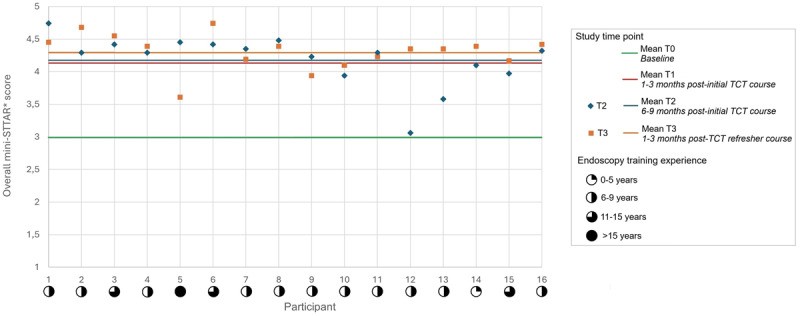
Impact of the Training the Colonoscopy Trainers (TCT) refresher training on individual trainer performances in the workplace.

Appendix 7 presents the mini-STTAR* outcomes from the video-recorded endoscopy training sessions conducted before (T2) and after (T3) implementation of the refresher training. Following training completion, participants demonstrated a significant improvement in the Set item ‘determines background knowledge’, with a medium effect size. No significant differences were observed for other mini-STTAR* items. Despite the lack of statistical significance for these items, a positive trend was evident at T3, with overall mini-STTAR*, Set, Dialogue, and Closure scores exceeding those at any other point in this study and in the prior study [4]. Figure 1 shows participants’ individual trainer performance at T2 and T3, alongside their endoscopy training experience.

### Kirkpatrick level 4

The post-refresher training resident survey was completed by six of the seven gastroenterology residents who had started formal endoscopy training (86% response); three women and three men. The group represented 33%, 50%, and 17% of residents in gastroenterology residency years three, four, and five, respectively.

All residents were satisfied with the supervision provided by endoscopy trainers at their teaching hospitals and perceived them as competent in teaching GI endoscopy skills (Appendix 8). Although a higher percentage of residents (strongly) agreed that pre-procedural briefings and post-procedural debriefings with endoscopy trainers were conducted after the TCT refresher training – an observation also reported in the free-text responses – there was no statistically significant difference compared to the pre-training survey results. Most residents reported improvements in spontaneous feedback and better guidance during difficult endoscopies.

## Critical Reflection on Process

This study is, to our knowledge, the first to develop and evaluate a refresher training specifically aimed at supporting the maintenance of procedural teaching skills. Both study objectives were achieved: the TCT refresher training supported sustained, high-quality endoscopy training for residents, and the evaluation identified key facilitators and barriers influencing the retention of endoscopy teaching skills in clinical practice. Reflecting on the development and implementation process, several strengths and challenges emerged.

A major strength was the evidence-informed educational design of the refresher training. Core elements of the original TCT course – previously shown to improve both endoscopy training quality [4] and patient outcomes [2] – were retained and reinforced, while additional components were introduced to support individual trainer performance over time. The structured training framework of Set, Dialogue, and Closure, comparable to the ‘Briefing, Intraoperative Teaching, and Debriefing’ model for effective learning in the operating room [18], provided a clear structure for teaching and was consistently applied in clinical practice, as reflected by mini-STTAR* scores (Appendix 7). In addition, the refresher training was grounded in experiential [13] and situated learning [19] principles, enabling trainers to practice and refine teaching skills within their authentic clinical context, supported by feedback and reflection.

Another strength was the comprehensive evaluation strategy. Although response tendencies (e.g. acquiescence bias or central tendency bias) cannot be fully excluded, survey data (Kirkpatrick level 1 and 4) were complemented by video-based analysis of endoscopy training in the workplace (Kirkpatrick level 3). This triangulation strengthens the validity of our findings and directly addresses calls in the FD literature to evaluate outcomes at higher Kirkpatrick levels, providing insight into the educational and organizational impact of FD initiatives [10,11,20].

Despite these strengths, several challenges emerged. Evidence from classroom-based teacher training indicates cognitive decline after approximately 200 days, suggesting that reinforcement at this point may be beneficial [6]. Somewhat unexpectedly, our previous evaluation of the original TCT course demonstrated higher mini-STTAR* scores at 6–9 months post-course (T2) than at 1–3 months (T1), indicating sustained – and possibly continued – improvement [4]. Although post-refresher training mini-STTAR* scores (T3) in this study exceeded all earlier measurements, statistically significant improvements were limited, likely reflecting the high performance 6–9 months after the original TCT course. Taken together, these findings raise important questions about the optimal timing of reinforcement and underscore the need for long-term follow-up studies to better understand the durability of procedural teaching skills.

The interviews highlighted the critical role of contextual and organizational factors in sustaining teaching quality. Institutional support emerged as a key facilitator, consistent with previous research [11,20]. Dedicated time for briefings (Set) between trainers and residents facilitated consistent application, whereas lack of time and limited opportunities for endoscopy training hindered development of effective supervision. Teaching and supervision should therefore be explicitly recognized as organizational priorities.

Should refresher training be considered a standard element of FD for procedural teaching? Our findings demonstrate its measurable value, even when the initial program has already achieved substantial impact. By “showing” how a refresher intervention strengthened and sustained clinician-educators’ teaching skills, this study “tells” why longitudinally embedded training may enhance skill retention, support transfer to procedural teaching, and contribute to cultural change within clinical environments. Refresher training should therefore not be considered remedial but as an integral component of sustained FD. We therefore advocate for its broader implementation and rigorous evaluation across FD initiatives beyond endoscopy.

## Additional File

The additional file for this article can be found as follows:

10.5334/pme.2443.s1 Appendices. Appendix 1 to 8.
