# Supplementary material for: Time to Refresh: Design and Evaluation of Refresher Training to Sustain Procedural Teaching Skills
Source: Perspect Med Educ. 2026 May 15;15(1):442–8. doi: 10.5334/pme.2443 (PMC13178612; doi:10.5334/pme.2443)
Supplement: Appendices. — Appendix 1 to 8. [file pme-15-1-2443-s1.pdf]

Appendix 1 – TCT refresher training interview questions

1. How do you currently perceive your role as an endoscopy trainer?
2. What learning objectives did you set for yourself at the conclusion of the TCT course?
3. Since completing the TCT course, have you changed any aspects of your approach to teaching endoscopy? If so, could you please specify how you implemented these changes?
4. What areas do you identify as opportunities for improvement in your approach to teaching endoscopy?
5. Are there any aspects of your teaching approach to endoscopy training that you would like to improve but find difficult to change? If so, could you please specify the reasons?
6. What are your personal learning goal(s) for the hands-on session during the refresher training, in which you will train a resident performing a colonoscopy on a patient?

Appendix 2 – Contextual study information

Endoscopy training in the Netherlands

This study was conducted in the northeastern educational region of the Netherlands, in which one university hospital (University Medical Center Groningen) and two affiliated general teaching hospitals (Isala Zwolle and Medisch Spectrum Twente) collaborate in providing a joint gastroenterology residency curriculum. Each year, four to five medical residents start their gastroenterology residency in this region.

In the Netherlands, the duration of gastroenterology residency varies between 65 and 72 months, depending on the residents' individual competencies. Although the training schedules of individual residents may differ, most residents complete their first two years of gastroenterology residency in a general teaching hospital and the final two years in a university hospital. Following 20 months of internal medicine training, residents usually start performing basic endoscopic procedures on patients in the third year of their residency. According to the national gastroenterology curriculum, the level of autonomy granted to residents in performing endoscopies is determined by the five Entrustable Professional Activities (EPA) levels of supervision, based on Direct Observation of Procedural Skills (DOPS) assessments. In the first phase of endoscopy training, residents learn to perform endoscopic procedures under the direct supervision of trainers (EPA level 2). During this phase, trainers are present in the endoscopy room throughout the entire procedure. After achieving a specified level of proficiency, residents progress to performing procedures under indirect supervision (EPA level 3) and eventually to supervision on request (EPA level 4). In these latter phases, trainers are not always present in the endoscopy room. A previous mixed-methods study among Dutch endoscopy trainers revealed considerable variability in training practices across gastroenterology teaching hospitals, including differences in the number of residents supervised simultaneously, and the presence of trainers during indirect supervision of more experienced residents [8]. Residents receive endoscopy training from multiple endoscopy trainers in their teaching hospitals.

The initial TCT course

Following concerns about suboptimal quality outcomes in colorectal cancer screening programs in the UK, a series of interventions were introduced within the UK's National Health Service to improve endoscopy training [2]. One such intervention was the development and implementation of the

Training the Colonoscopy Trainers (TCT) course, grounded in the principle that proficiency in performing endoscopy does not automatically translate into effective teaching, and that formal training in procedural teaching is required [2]. Several years after its implementation, colonoscopy quality indicators in the UK improved, and the TCT course was adopted internationally [2]. In 2018, the first TCT courses in the Netherlands were delivered at Leiden University Medical Center [4]. This Dutch adaptation of the original TCT course [2] was facilitated by faculty trained at St. Mark's Hospital in the UK [4]. The two-day course, accommodating up to six participants, integrates pedagogical theory (e.g. developing conscious competence, applying a structured training framework, preventing cognitive overload, and delivering performance-enhancing feedback) with practical endoscopy teaching skills [2]. The first day consists of interactive theoretical sessions addressing key principles of endoscopy teaching, supported by the use of a virtual endoscopy simulator and a colonoscopy training model. The second day focuses on hands-on teaching practice: participants supervise residents performing colonoscopies on actual patients, applying the instructional principles learned at day one. These sessions take place in the endoscopy room, with one faculty member present in the room, and the other faculty member(s) and other participants observing via a livestream from an adjacent room [4]. Unlike in the UK [2], formal training in endoscopy teaching is not mandatory for endoscopy trainers in the Netherlands [8].

Appendix 3 – Post-training questionnaire

Demographics

1. Gender (m/f)
2. The number of years I have been supervising residents in the endoscopy department is ... (number)

TCT course

3. Following the TCT course, I feel more competent in supervising residents in the endoscopy department (5-point Likert scale)
4. Following the TCT course, I enjoy supervising residents in the endoscopy department more (5-point Likert scale)
5. Following the TCT course, I take over the endoscope less often when a resident encounters difficulties (5-point Likert scale)
6. Following the TCT course, I more frequently attend the entire procedure when a resident performs an endoscopy under indirect supervision (EPA level 3/4) (5-point Likert scale)
7. Following the TCT course, I more frequently discuss the patients with the resident prior to the endoscopy list (5-point Likert scale)
8. Following the TCT course, I more frequently debrief the patients with the resident after the endoscopy list (5-point Likert scale)
9. Following the TCT course, I more frequently set learning objectives with the resident prior to the endoscopy list (5-point Likert scale)
10. Following the TCT course, I more frequently evaluate with the resident after the endoscopy list whether the predefined learning objectives have been achieved (5-point Likert scale)
11. Following the TCT course, I more frequently complete a DOPS assessment with the resident (5-point Likert scale)
12. My approach to supervising residents in the endoscopy department has changed following the completion of the TCT course (5-point Likert scale)
13. The most important changes in endoscopy supervision since the TCT course, from my personal perspective, are: (free text)

14. The most important changes in endoscopy supervision at the staff/department level since the TCT course are: (free text)
15. After the initial TCT course, I participated in the TCT refresher training (yes/no)

TCT refresher training

16. Participating in the TCT refresher training following completion of the initial TCT course is valuable (5-point Likert scale)
17. Participation in the TCT refresher training following the initial TCT course should become standard practice (5-point Likert scale)
18. Please provide a brief explanation for your responses to the two questions above (free text)

Appendix 4 – Adapted mini-Structured Training Trainer Assessment Report (mini-STTAR*)

Mini-STTAR*

**EPA level resident**:	**Case**:	**Type of endoscopy**:

SCORE (degree to which training item occurred):
1. Did not happen but should have been done
2. Happened but not enough
3a. Did not happen but did not need to
3b. Happened but did not need to
4. Happened perfect amount
N/A. Not applicable

	TRAINING ASSESSMENT	1	2	3a	3b	4	N/A
SET	1. Determines background knowledge	◻	◻	◻	◻	◻	
	2. Defines aims	◻	◻	◻	◻	◻	
	3. Discusses case specific information	◻	◻	◻	◻	◻	
	4. Aligns agendas	◻	◻	◻	◻	◻	
	5. Establishes groundrules	◻	◻	◻	◻	◻	
	6. Communicates with team	◻	◻	◻	◻	◻	
	7. Ensures patient safety	◻	◻	◻	◻	◻	
TRAINING	8. Guiding/deconstructing, e.g. 'the next step is to…'	◻	◻	◻	◻	◻	
	9. Directing, e.g. 'go in a bit… lift up'	◻	◻	◻	◻	◻	
	10. Questioning, e.g. 'what would you do next'	◻	◻	◻	◻	◻	
	11. Clarifying, e.g. 'why are you doing that'	◻	◻	◻	◻	◻	
	12. Encouraging/praise	◻	◻	◻	◻	◻	
	13. Informing, e.g. general information about disease	◻	◻	◻	◻	◻	
	14. Corrective feedback	◻	◻	◻	◻	◻	
	15. Warning, e.g. 'if you do that you'll injure the bowel'	◻	◻	◻	◻	◻	
	16. Controlling, e.g. 'stop'	◻	◻	◻	◻	◻	
	17. Pushes trainee – takes them out of the comfort zone	◻	◻	◻	◻	◻	
	18. Calm	◻	◻	◻	◻	◻	
	19. Patient	◻	◻	◻	◻	◻	
	20. Comfortable in silence	◻	◻	◻	◻	◻	
	21. Non-threatening	◻	◻	◻	◻	◻	
	22. Communicates clearly	◻	◻	◻	◻	◻	
	23. Takes over when necessary	◻	◻	◻	◻	◻	
CLOSURE	24. Critiques performance	◻	◻	◻	◻	◻	
	25. Asks trainees opinion	◻	◻	◻	◻	◻	
	26. Encourages reflection	◻	◻	◻	◻	◻	
	27. Honest	◻	◻	◻	◻	◻	
	28. Approachable (allows discussion)	◻	◻	◻	◻	◻	
	29. Seeks feedback	◻	◻	◻	◻	◻	
	30. Behaves like an excellent role model	◻	◻	◻	◻	◻	
	31. Develops learning point agreement	◻	◻	◻	◻	◻	

	Strongly disagree	Disagree	Undecided	Agree	Strongly agree
Overall is an excellent trainer	◻	◻	◻	◻	◻

Summary comments:

Appendix 5 – Post-training resident survey

Demographics

1. Gender (m/f)
2. Current gastroenterology residency year (3, 4, 5 or 6)

Perceived quality of endoscopy supervision

3. I am satisfied with the quality of endoscopy supervision at my teaching hospital (5-point Likert scale)
4. Endoscopy trainers at my teaching hospital are competent in teaching gastrointestinal endoscopic skills (5-point Likert scale)
5. Endoscopy trainers at my teaching hospital provide uniform endoscopy supervision (5-point Likert scale)
6. Sometimes I exceed my capacity during endoscopic procedures (5-point Likert scale)
7. Before the start of an endoscopy training session, I discuss the patients on the list with the endoscopy trainer (5-point Likert scale)
8. Before the start of an endoscopy training session, I set learning objectives with the endoscopy trainer (5-point Likert scale)
9. After an endoscopy training session, I debrief the training with the endoscopy trainer and identify areas for improvement (5-point Likert scale)
10. After an endoscopy training session, trainers are prepared to complete a Direct Observation of Procedural Skills (DOPS) evaluation (5-point Likert scale)

TCT refresher training

11. The TCT refresher training has led to an improvement in endoscopy supervision at my teaching hospital (5-point Likert scale)
12. Since the implementation of the TCT refresher training, endoscopy trainers encourage me more to set a specific learning objective before an endoscopy training session (5-point Likert scale)
13. Since the implementation of the TCT refresher training, endoscopy trainers more frequently discuss whether a previously set learning objective has been achieved after an endoscopy training session (5-point Likert scale)

14. Since the implementation of the TCT refresher training, endoscopy trainers more frequently provide spontaneous feedback during an endoscopy training session (5-point Likert scale)
15. Since the implementation of the TCT refresher training, endoscopy trainers are better able to guide me during a difficult endoscopy (5-point Likert scale)
16. Since the implementation of the TCT refresher training, endoscopy trainers are less likely to take over the endoscope without consent during a difficult endoscopy (5-point Likert scale)

Open ended questions

17. The main positive points of the TCT refresher training on the endoscopy supervision of gastroenterologists at my teaching hospital are (free text)
18. The main negative effects of the TCT refresher training on the endoscopy supervision of gastroenterologists at my teaching hospital are (free text)

Appendix 6 – Kirkpatrick level 1 outcomes (reaction)

Participants' perspectives regarding faculty development programs focusing on endoscopy teaching	
	Number (%) of respondents that agreed (Likert scale 4-5) with the statement (N=12)
Initial TCT course improved competence in endoscopy training	12 (100)
Initial TCT course enhanced satisfaction in endoscopy training	10 (83)
Initial TCT course reduced frequency of endoscope takeover	6 (50)
Initial TCT course improved frequency of pre-procedural briefing	8 (67)
Initial TCT course improved frequency of post-procedural debriefing	8 (67)
Initial TCT course improved frequency of setting learning objectives	10 (83)
Initial TCT course improved frequency of evaluating learning objectives	10 (83)
Initial TCT course improved frequency of completing DOPS assessment	5 (42)
TCT refresher training participation following initial TCT course is valuable	12 (100)
TCT refresher training participation should become standard practice	10 (83)

Descriptive statistics; N = number of participants; DOPS = Direct Observation of Procedural Skills; TCT = Training the Colonoscopy Trainers

Appendix 7 – Kirkpatrick level 3 outcomes (behaviour)

Impact of the TCT refresher training on trainer performance in the workplace				
**Mini-STTAR* items**	**T2**<br>**Mean score (SD)**	**T3**<br>**Mean score (SD)**	**Effect size**	**P-value**
**Overall mini-STTAR* score**	4.18 (0.40)	4.31 (0.28)		0.244
**Set**				
1. Determines background knowledge	2.50 (2.00)	3.81 (1.83)	0.51	0.040*
2. Defines aims	4.75 (1.00)	4.75 (1.00)		
3. Discusses case specific information	2.88 (1.96)	3.31 (1.99)		0.453
4. Aligns agendas	4.75 (1.00)	4.75 (1.00)		
5. Establishes ground rules	2.50 (2.00)	3.00 (2.07)		0.317
6. Communicates with team	3.00 (0.00)	3.00 (0.00)		
7. Ensures patient safety	5.00 (0.00)	5.00 (0.00)		
Overall Set score	3.63 (0.70)	3.95 (0.75)		0.124
**Dialogue**				
8. Guiding/deconstructing	4.00 (1.79)	3.63 (1.86)		0.746
9. Directing	5.00 (0.00)	5.00 (0.00)		
10. Questioning	4.75 (1.00)	5.00 (0.00)		0.317
11. Clarifying	5.00 (0.00)	5.00 (0.00)		
12. Encouraging/praise	5.00 (0.00)	4.81 (0.75)		0.317
13. Informing	5.00 (0.00)	5.00 (0.00)		
14. Corrective feedback	4.62 (0.81)	4.62 (0.81)		
15. Warning	3.25 (0.68)	3.38 (0.81)		0.655
16. Controlling (stop)	3.31 (1.99)	3.81 (1.83)		0.216
17. Pushes trainee	3.75 (1.61)	3.81 (1.33)		0.957
18. Calm	5.00 (0.00)	5.00 (0.00)		
19. Patient	5.00 (0.00)	5.00 (0.00)		
20. Comfortable in silence	5.00 (0.00)	5.00 (0.00)		
21. Non-threatening	5.00 (0.00)	5.00 (0.00)		
22. Communicates clearly	5.00 (0.00)	5.00 (0.00)		
23. Takes over when necessary	3.81 (0.98)	4.06 (1.00)		0.414
Overall Dialogue score	4.53 (0.26)	4.57 (0.29)		0.681
**Closure**				
24. Critiques performance	4.75 (1.00)	5.00 (0.00)		0.317

25. Asks trainees opinion	4.75 (1.00)	5.00 (0.00)	0.317
26. Encourages reflection	3.31 (1.99)	3.56 (1.93)	0.660
27. Honest	4.75 (1.00)	5.00 (0.00)	0.317
28. Approachable (allows discussion)	4.75 (1.00)	5.00 (0.00)	0.317
29. Seeks feedback	1.00 (0.00)	1.00 (0.00)	
30. Behaves like an excellent role model	4.75 (1.00)	5.00 (0.00)	0.317
31. Develops learning point agreement	3.75 (1.92)	3.25 (2.05)	0.414
Overall Closure score	3.98 (0.89)	4.10 (0.37)	0.809

Statistics: Wilcoxon signed-rank test, significant difference (*P*<0.05); SD = standard deviation; T2 = pre-TCT refresher training; T3 = post-TCT refresher training

Appendix 8 – Kirkpatrick level 4 outcomes (results)

Gastroenterology residents' perspectives regarding endoscopy supervision before and after the TCT refresher training			
	Pre-TCT refresher training survey (N=11) Number (%) of participants that agreed (Likert scale 4-5) with the statement	Post-TCT refresher course training (N=6) Number (%) of participants that agreed (Likert scale 4-5) with the statement	P-value
Satisfied with current endoscopy supervision	11 (100)	6 (100)	
Trainers are competent in teaching endoscopic skills	10 (91)	6 (100)	.446
Uniform endoscopy supervision among trainers	5 (45)	4 (67)	.402
Application of pre-procedural briefing (Set)	5 (45)	5 (83)	.129
Application of post-procedural debriefing (Closure)	2 (18)	3 (50)	.169
DOPS evaluation after training	10 (91)	4 (67)	.210
TCT refresher training improved endoscopy supervision		3 (50)	
TCT refresher training improved setting learning objectives		2 (33)	
TCT refresher training improved evaluating learning objectives		0 (0)	
TCT refresher training improved spontaneous feedback		4 (67)	
TCT refresher training improved difficult endoscopy guidance		4 (67)	
TCT refresher training improved undesired scope takeover		1 (17)	

Statistics: χ^2-test; N = number of participants; DOPS = Direct Observation of Procedural Skills; TCT = Training the Colonoscopy Trainers
